# The Reprimo gene family member, *reprimo-like* (*rprml*), is required for blood development in embryonic zebrafish

**DOI:** 10.1038/s41598-019-43436-8

**Published:** 2019-05-09

**Authors:** Karen Stanic, German Reig, Ignacio A. Wichmann, Juan C. Opazo, Gareth I. Owen, Alejandro H. Corvalán, Miguel L. Concha, Julio D. Amigo

**Affiliations:** 1 Departamento de Fisiología, Facultad de Ciencias Biológicas, Pontificia Universidad Católica de Chile, Santiago, Chile; 2 Institute of Biomedical Sciences, Faculty of Medicine, Universidad de Chile, Santiago, Chile; 3 Advanced Center for Chronic Diseases (ACCDiS), Santiago, Chile; 4 Laboratorio de Oncología, Departamento de Hematología y Oncología, Facultad de Medicina, Pontificia Universidad Católica de Chile, Santiago, Chile; 5 Instituto de Ciencias Ambientales y Evolutivas, Facultad de Ciencias, Universidad Austral de Chile, Valdivia, Chile; 6 Millennium Institute on Immunology and Immunotherapy, Santiago, Chile; 7 Biomedical Neuroscience Institute, Santiago, Chile, Center for Geroscience, Brain Health and Metabolism, Santiago, Chile; 8 Universidad Bernardo O´Higgins, Escuela de Tecnología Médica and Centro Integrativo de Biología y Química Aplicada (CIBQA), Santiago, Chile

**Keywords:** Leukopoiesis, Genetics research, Haematopoiesis

## Abstract

The *Reprimo* gene family comprises a group of single-exon genes for which their physiological function remains poorly understood. Heretofore, mammalian *Reprimo* (*RPRM*) has been described as a putative p53-dependent tumor suppressor gene that functions at the G2/M cell cycle checkpoint. Another family member, *Reprimo-like* (*RPRML*), has not yet an established role in physiology or pathology. Importantly, *RPRML* expression pattern is conserved between zebrafish and human species. Here, using CRISPR-Cas9 and antisense morpholino oligonucleotides, we disrupt the expression of *rprml* in zebrafish and demonstrate that its loss leads to impaired definitive hematopoiesis. The formation of hemangioblasts and the primitive wave of hematopoiesis occur normally in absence of *rprml*. Later in development there is a significant reduction in erythroid-myeloid precursors (EMP) at the posterior blood island (PBI) and a significant decline of definitive hematopoietic stem/progenitor cells (HSPCs). Furthermore, loss of *rprml* also increases the activity of caspase-3 in endothelial cells within the caudal hematopoietic tissue (CHT), the first perivascular niche where HSPCs reside during zebrafish embryonic development. Herein, we report an essential role for *rprml* during hematovascular development in zebrafish embryos, specifically during the definitive waves of hematopoiesis, indicating for the first time a physiological role for the *rprml* gene.

## Introduction

Early stages of vertebrate hematopoiesis involve two developmental phases, defined as primitive and definitive hematopoiesis. In zebrafish, the primitive wave of hematopoiesis starts from two stripes of hemangioblast-like cells positioned in the lateral plate mesoderm at early somitic stages: i) the anterior lateral-plate mesoderm (ALM), which gives rise to myeloid cells, and ii) the posterior lateral-plate mesoderm (PLM), which produces the primitive erythroid cells^1,2^. PLM cells migrate across the midline to originate the intermediate cell mass (ICM), where the primitive erythroid cells complete their maturation between the nascent dorsal aorta (DA) and posterior cardinal vein plexus (PCVP). By 26 hours post-fertilization (hpf), the first erythrocytes begin to circulate. This process occurs together with the expression of the proto-oncogene transcription factor *cmyb* in the first hematopoietic cells budding from the ventral DA (VDA)^3,4^.

In zebrafish, definitive hematopoiesis also occurs in two waves. The first wave, named transient-definitive hematopoiesis produces erythroid-myeloid progenitors (EMP) in the posterior blood island (PBI)^5^. The second wave is characterized by the generation of hematopoietic stem/progenitor cells (HSPCs) which can either undergo self-renewal or differentiate into erythroid, myeloid and lymphoid hematopoietic lineages^1,2^. HSPCs, induced by somite-derived endothelial cells^6^, arise in the VDA in a region also known as the aorta-gonad-mesonephros (AGM) and characteristically express *cmyb* and/or *runx1* after 26 hpf^7,8^. The HSPCs emerge from the hemogenic endothelium in the floor of the AGM or VDA, through a process termed endothelial hematopoietic transition (EHT). Previous studies using *CD41:GFP*^*low*^ transgenic animals have defined the migration of HSPC from the AGM to their niche in the caudal hematopoietic tissue (CHT) and then to thymus and pronephros^3,9^. Recent evidence by our group has reported the expression pattern of the *RPRM* gene family members during blood vessel development^10^.

The *Reprimo* (*RPRM*) gene family is a group of single-exon genes exclusive to the vertebrate group, that diversified as a product of two rounds of whole genome duplications occurred in the last common ancestor of vertebrates between 676 and 615 million years ago^10–12^. The repertoire of *Reprimo* genes includes *Reprimo* (*RPRM*), *Reprimo-like* (*RPRML*) and *Reprimo 3* (*RPRM3*). To date, only the human *RPRM* gene has been studied^10,12^, with no literature describing the role of *RPRML* or *RPRM3* in either physiology or pathophysiology. In humans, *RPRM* is a highly glycosylated cytoplasmic protein, which has been characterized as a potential tumor-suppressor gene involved in the regulation of the *p53*-mediated cell cycle arrest at G2 through the regulation of the Cdc2-cyclin B1 complex activity by a yet undiscovered mechanism^13^.

Zebrafish (*Danio rerio*) have two co-orthologs of the mammalian *RPRM* gene (designated *rprma*, *rprmb*) and a single co-ortholog of *RPRML* (*rprml*) and *RPRM3* (*rprm3*), all of which are expressed during normal embryogenesis^10,14^. Gene expression profiling of the *rprm* genes suggested that during their evolutionary history, they have undergone a process of subfunctionalization with some degree of redundancy, as they exhibit unique – although partially overlapping – expression profiles during embryonic and larval blood vessel development. Interestingly, epigenetic silencing of *RPRM* has been linked to human blood cancers such as pediatric myeloid leukemia^15^. These studies suggest that *RPRM* genes may be involved in the formation and differentiation of the blood lineages in a process known as hematopoiesis.

Herein, we characterize the functions of *rprml* during hematovascular development in zebrafish. By loss-of-function assays either using generated knockdown CRISPR mutants (G0) or morphant embryos, we demonstrate that *rprml* is required during both waves of definitive hematopoiesis for the specification of both EMP and HSPC. Furthermore, we report that *rprml* regulates the normal formation of the HSPC perivascular PCVP/CHT niche. To our knowledge, this is the first report describing a physiological role for *rprml*.

## Results

### *rprml* loss-of-function using MO and CRISPR-Cas9 systems

We previously reported distinctive RNA expression pattern profiles for *RPRM* gene family members^10,14^. We also showed that *rprml* embryonic expression domains include vascular and mesodermal-derived tissues. Here, to assess a possible function of *rprml* during blood development, we used a combination of antisense oligonucleotide morpholinos (MOs)^16^ and CRISPR-Cas9^17^ genetic approaches in a transgenic *Tg*(*fli1:GFP*) background, which enabled us to visualize endothelial cells (EC) by confocal microscopy. After comparing uninjected WT embryos with MO-injected and mutant specimens, where we observed no evidence of developmental delay generated by the injection (Fig. S1), we compared the phenotypes between the embryos injected with MOs targeting *rprml* and the CRISPR-Cas9 G0 *rprml* mutants^17^. The observed phenotypes were comparable in both experiments (Fig. S2A–C), characterized by reduced CHT territory and mild alterations in vascular morphology at the PCVP, which did not affect normal blood flow. Additionally, macroscopic observations of the different specimens showed no apparent decrease in the length of the trunk (Fig. S1). Abnormal blood accumulation in yolk, trunk edema and or hemorrhage were absent in CRISPR-Cas9 and morphant specimens. Cranial vasculature was also normal in controls compared with morphant and mutant embryos (Fig. S1).

In accordance with the recent guidelines and standard use of MO in zebrafish embryos^18^, we assessed the expression of *p53*^19,20^ by qRT-PCR in MO-injected and Tab5 WT uninjected embryos. This control was performed to rule out activation of p53 and associated cell death in MO-injected embryos. We observed a moderate decrease in p53 expression levels in a pool of *rprml*-MO injected specimens compared with the control group (Fig. S2G), supporting that this MO dosage was not toxic to the embryos. To assess the effectiveness of the *rprml* MO knockdown approach, we performed immunohistochemical analysis of Rprml protein. We observed a significant reduction of Rprml in *rprml*-MO embryos when compared with controls (Fig. S2D,E). Additionally, we performed phenotypic rescue experiments to assess the specificity of the MO. Co-injection of *rprml* mRNA and *rprml*-MO renders significantly rescued *rprml-*MO phenotype (Fig. S5), indicating that the *rprml*-MO effects over hematopoiesis are specific. Taken together, these results and the comparable phenotypes obtained by antisense MO and CRISPR-Cas9 genetic approaches, validate the use of MOs for the assessment of the physiological role of *rprml* during zebrafish embryogenesis by gene loss-of-function.

### *rprml* is dispensable for primitive hematopoiesis but required for HSPC development

**Figure 1:**
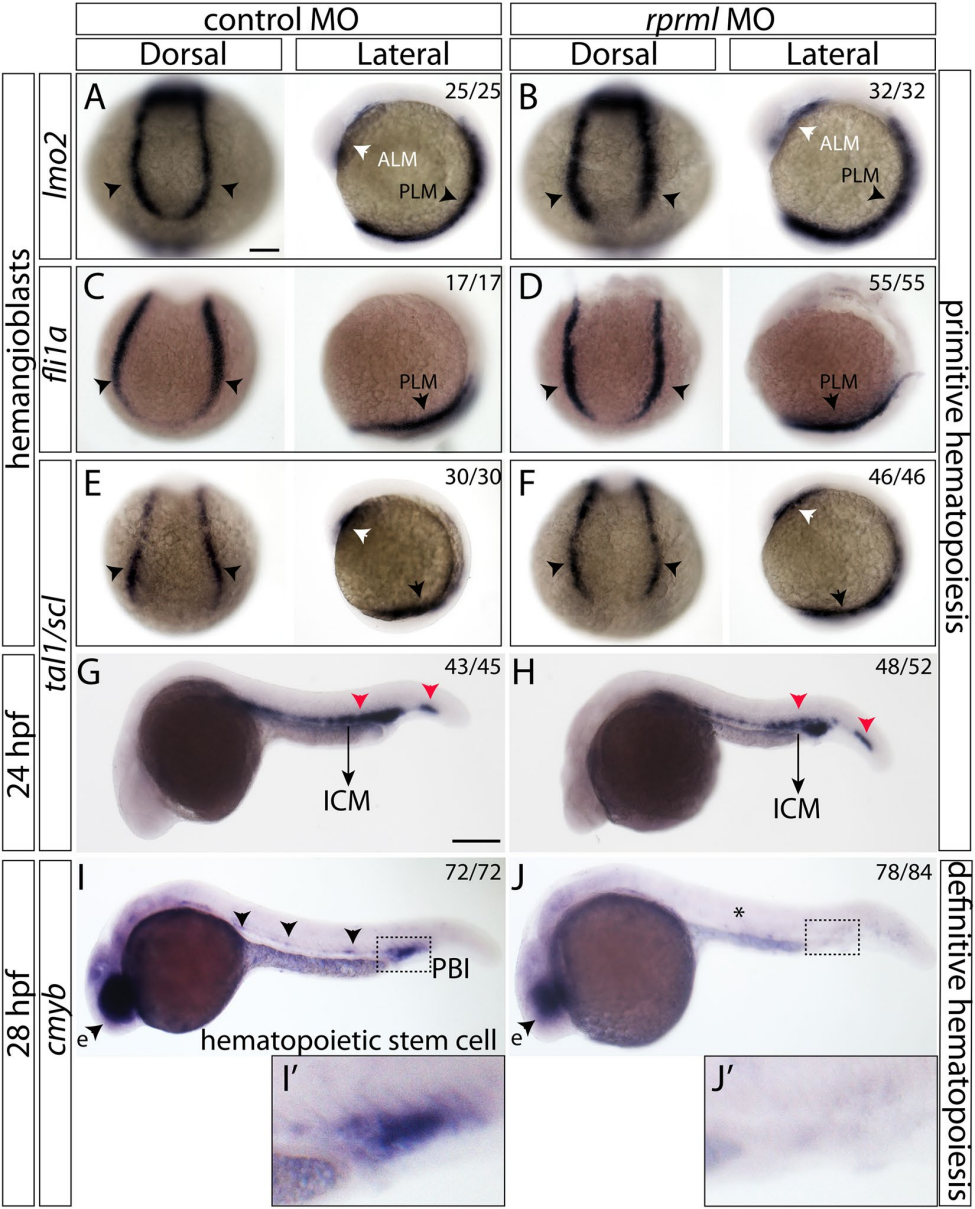
*rprml* is dispensable for primitive hematopoiesis but required for HSPC formation. (**A**–**F**) Dorsal and lateral views of 10 somite-stage zebrafish embryos analyzed by WISH. White and black arrowheads represent the anterior- (ALM) and posterior-lateral plate mesoderm (PLM), respectively. (**A**,**C**,**E**,**G**,**I**) control MO-injected embryos; (**B**,**D**,**F**,**H**,**J**) *rprml* MO-injected embryos. (**A**–**F**) Expression patterns of typical hemangioblast markers: (**A**,**B**) *lmo2*, (**C**,**D**) *fli1a*, (**E**,**F**) *tal1/scl1*. (**G**,**H**) Lateral view of 24 hpf control MO and *rprml* MO-injected embryos stained by WISH for *tal1/scl1*. Black arrows indicate the intermediate cell mass (ICM) and red arrowheads normal expression pattern for *tal1/scl1*. (**I**,**J**) 28 hpf embryos stained by WISH for *cmyb* comparing controls versus *rprml* MO-injected embryos. Black arrowheads indicate normal expression pattern for *cmyb*. *rprml* morphant embryos retain retinal expression of *cmyb* (eye, e). The number of embryos with the phenotype shown as a fraction of the total number of embryos examined is indicated in the top right corner in (**A**–**J**). Scale bars, 100 μm (**A**–**F**) and 200 μm (**G**–**J**).

At early developmental stages, hemangioblasts contain the common precursor cells for hematopoietic and vascular lineages at the anterior and posterior lateral plate mesoderm (ALM and PLM)^1,2^. We used whole mount *in situ* hybridization (WISH) to determine the expression of well-known hemangioblast molecular markers in control and morphant embryos at 5–10 somite-stage, including *lmo2*, *fli1a* and *tal1/scl1*. We found comparable expression levels of these markers, suggesting that *rprml* does not play a relevant role during cell specification in primitive hematopoiesis (Fig. 1A–F). By 22 hpf, expression of *tal1/scl1* (a primitive erythroid marker) remained mostly unaffected at the intermediate cell mass (ICM) (Fig. 1G,H). At around 24 hpf, we did not detect significant changes in the expression of *lmo2* in *rprml* morphants compared with control embryos (Fig. S3A,B). However, we did observe a moderate reduction of *fli1a* in the PCVP at the CHT (Fig. S3C,D). In contrast, after 26 hpf, abrogation of *rprml* function led to a drastic reduction in the *cmyb*^+^ HSPCs population at the ICM and the posterior blood island (PBI) (Fig. 1I,J,I′,J′ in detail). At the same developmental stage, the expression of *cmyb* remained unaltered in neuronal territories such as the retina in *rprml* morphants (Fig. 1I,J, arrowheads). Therefore, loss-of-function of *rprml* does not alter hemangioblast in the ALM/PLM or primitive hematopoiesis but is likely required for HSPCs formation.

### *rprml* is required for transient-definitive wave of hematopoiesis

**Figure 2:**
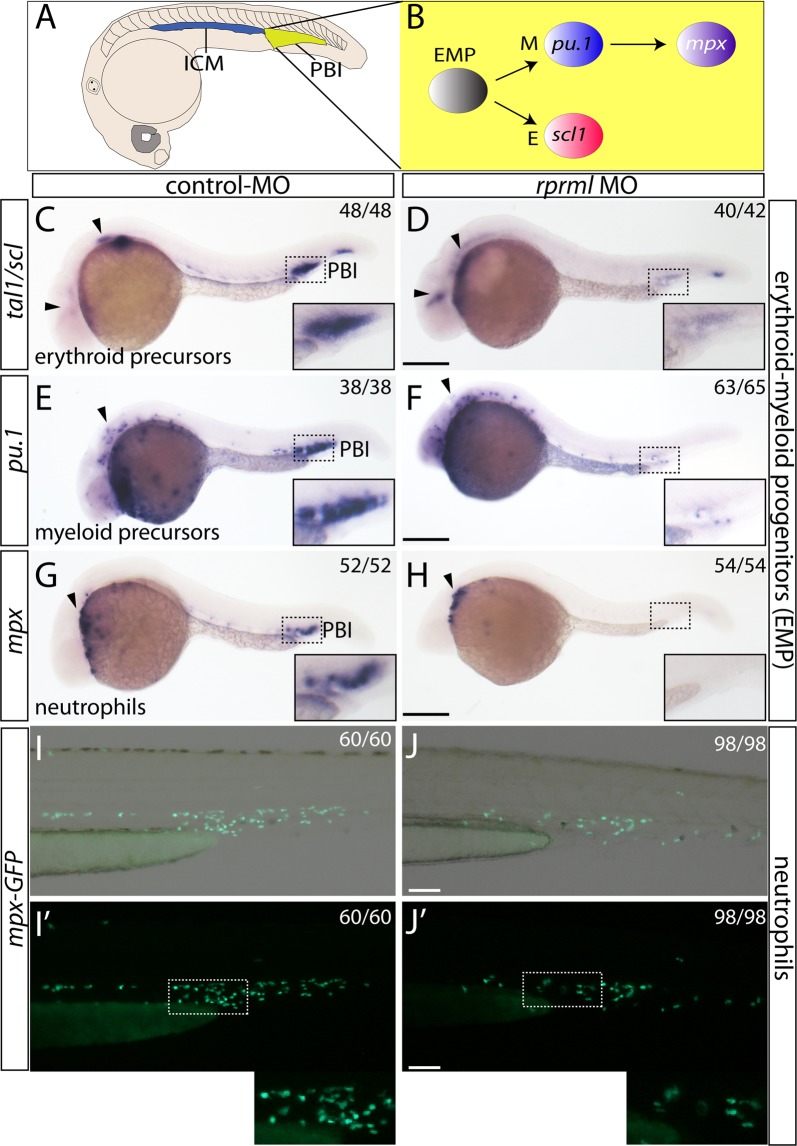
*rprml* is required for transient-definitive hematopoiesis. (**A**) Schematic diagram for zebrafish hematopoiesis at 28 hpf showing the intermediate cell mass (ICM) and the posterior blood island (PBI). (**B**) Schematic for the transient-definitive wave of hematopoiesis where EMP give rise to erythroid myeloid and granulocytic/neutrophils cell population at the PBI. (**C**,**E**,**G**,**I**) Lateral views of control MO and (**D**,**F**,**H**,**J**) *rprml* MO-injected embryos. (**C**–**H**) 28 hpf embryos analyzed by WISH against erythroid/myeloid progenitor and granulocyte markers: (**C**–**D**) *tal1/scl1* for erythroid precursor cells, (**E**,**F**) *pu*.*1* for myeloid precursor cell, (**G**,**H**) *mpx* for neutrophils. Inset magnifications from (**C**) to (**H**) show the posterior blood island (PBI). Black arrowheads in (**D**,**F**,**H**) show normal expression of *tal1/scl1*, *pu*.*1* and *mpx* in the anterior primitive hematopoietic territories. (**I**,**J**) Representative images of 48 hpf transgenic *Tg*(*mpx:GFP*) embryos analyzed by fluorescent microscopy. (**I**,**I′**) control and (**J**,**J′**) *rprml* morphant embryos. Inset magnifications in (**I′**,**J′**) show positive fluorescence in neutrophils at the caudal hematopoietic tissue. Number of embryos with the phenotype shown as a fraction of the total number of embryos examined is indicated in the top right corner in (**C**–**J**). Scale bars, 200 μm (**C**–**H**) and 100 μm (**I**,**J**).

As stated previously, definitive hematopoiesis occurs in two successive waves during zebrafish development^1^. The first transient-definitive wave involves the formation of erythroid-myeloid progenitors (EMP) at the PBI (Fig. 2A,B). The second definitive wave produces HSPCs from the hemogenic endothelium located in the AGM. Compared with HSPCs, EMP in zebrafish and mouse lack capacity for self-renewal^5^. To assess the possible functional role of *rprml* during transient-definitive hematopoiesis, we analyzed the expression pattern of the erythroid progenitor marker *tal1/scl1* by using WISH. We noted a significant reduction of gene expression at the PBI in *rprml*^*kd*^ (Fig. 2C,D), while normal expression was retained in the brain (compared Fig. 2C,D, arrowheads). Furthermore, expression of the myeloid progenitor marker *pu*.*1* was also reduced at the PBI of *rprml*^*kd*^ compared with control embryos (Fig. 2E,F). Of note, *pu*.*1* expression was unaffected in the primitive myeloid precursors of the rostral ICM (compared Fig. 2E,F, arrowheads). We also investigated the development of granulocytic cells in *rprml*^*kd*^. To this end, we analyzed the expression of the neutrophil marker *mpx* and noted reduced expression in *rprml*^*kd*^ when compared to control embryos (Fig. 2G,H). Also, the number of neutrophils was drastically reduced in the CHT of transgenic *Tg*(*mpx:GFP*) embryos between 32 and 48 hpf (Fig. 2I–J′), consistent with the decreased *mpx* expression visualized by WISH. Altogether, these results indicate that *rprml* is required for EMP formation during the transient wave of definitive hematopoiesis.

### Loss of *rprml* hinders HSPC specification

**Figure 3:**
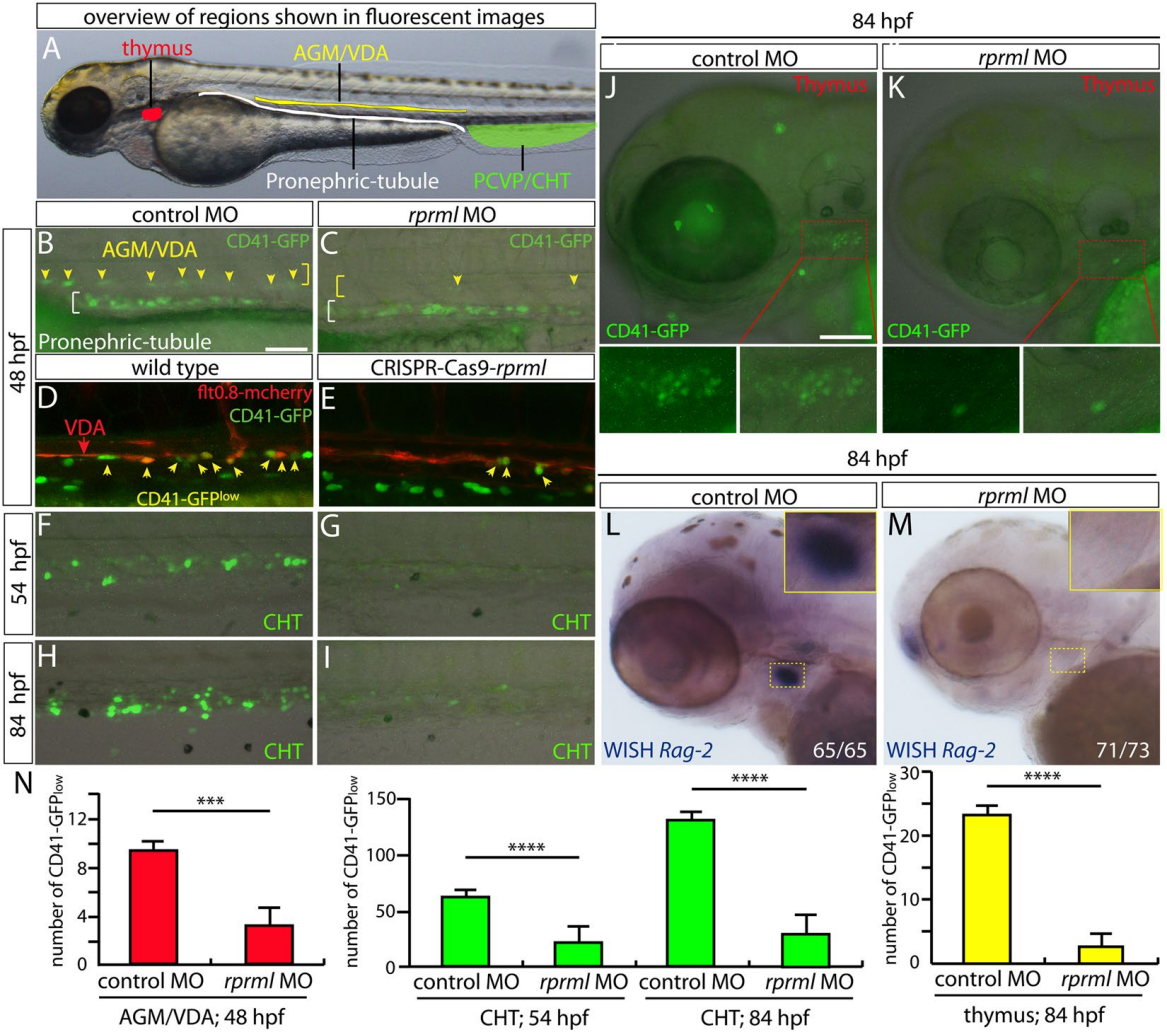
*rprml* is required for specification of hematopoietic precursor/stem cells (HSPCs) from the hemogenic endothelium at the ventral dorsal aorta. (**A**) Lateral view of zebrafish embryo with anterior to the left, showing overview of regions magnified in the fluorescent imaging (**B**–**K**) and WISH (**L**,**M**) panels. Red region denotes the left thymic lobe, yellow region the anterior gonad mesonephros/ventral dorsal aorta (AGM/VDA), green region the posterior caudal vein plexus/caudal hematopoietic tissue (PCVP/CHT) and white region the pronephric-tubule. (**B**–**M**) Lateral views of zebrafish embryos with anterior to the left at denoted developmental stages (48, 54 and 84 hpf). Loss-of function of *rprml* impairs normal formation of CD41-GFP^low+^ HSPCs (yellow arrows in **B**–**E**) originating from the AGM/VDA (**B**–**E**, red bar graphs in N) and their migration to the CHT (**F**–**I**, green bar graphs in N) and thymus (**J**,**K**, yellow bar graphs in N). (**D**,**E**) Double transgenic *Tg*(*flt0*.*8:mcherry;CD41:GFP*) CRISPR-Cas9-*rprml* injected embryos show the same decrease in HSPCs originating from the AGM/VDA as *rprml*-morphant embryos. (**L**,**M**) WISH showing that *rprml*-morphant embryos show a remarkable absence of *rag2* (lymphocyte marker) in the thymus. Statistical significance was determined using two-tailed unpaired Student’s *t*-test. ***P ≤ 0.001, ****P ≤ 0.0001. Scale bars, 50 μm (**B**–**I**) and 100 μm (**J**–**M**).

As mentioned above, zebrafish *rprml*^*kd*^ animals exhibited normal primitive hematopoiesis including specification of erythroid and myeloid cells (Fig. 1A–F). During definitive hematopoiesis, at 28 hpf, the *cmyb*^+^ HSPC population was greatly reduced in *rprml*^*kd*^ when compared with control embryos (Fig. 1I,J). Emergence of HSPCs from the ventral DA (VDA) has been characterized by the expression of *CD41:GFP*^*low*^ in the AGM (Fig. 3A). To visualize HSPC dynamics, we used time-lapse confocal imaging in double transgenic *Tg*(*flt0*.*8:mcherry;CD41:GFP*) and single *Tg*(*CD41:GFP*) embryos (Fig. 3). Consistent with our previous results, zebrafish *rprml*^*kd*^ showed a drastic reduction of *CD41:GFP*^*low*+^ HSPCs budding derived from the VDA (yellow arrows, Fig. 3B,C,N), a result that was also confirmed in CRISPR-Cas9-*rprml* mutants (Fig. 3D,E). Thus, the emergence of HSPCs from the AGM/VDA seems to be strongly dependent on *rprml* activity. Consequently, initial HSPCs migration to the CHT was substantially reduced at 54 hpf (Figs 3F,G,N and S4). This reduction in HSPCs number at the CHT persisted later in development, as the *rprml* morphants showed greatly reduced *CD41:GFP*^*low*+^ cells compared with control embryos at 84 hpf (Fig. 3H,I,N). In parallel, the dorsal-ventral width of the CHT was drastically reduced after 48 hpf. Additionally, in *rprml*-MO injected *Tg*(*CD41:GFP*) embryos, *CD41:GFP*^*low*+^ lymphocyte progenitors failed to colonize the thymus (Fig. 3J,K,N). Furthermore, *rag2*^+^ lymphocytes, which depend on upstream HSPCs^21^, were almost completely absent in *rprml* morphants (Fig. 3L,M). Finally, the number of *CD41*^+^*-GFP* and *mpx*^+^*-GFP* expressing cells was significantly restored by co-injection of zebrafish *rprml* mRNA in *rprml*-morphants, indicating that the effects over hematopoiesis by *rprml*-MO injections are specific (Fig. S5).

There is a close relationship between HSPC formation and the aortic endothelium^7,8^. To characterize arterial development, we performed WISH using several early arterial-specific markers. We examined the expression of two typical zebrafish artery markers, *kdr1* (also known as *vegfr-2*) and *dll4* (Notch ligand). Our results show that the expression of both markers did not drastically change in morphants when compared with control embryos (Fig. S6A–D). Consistently, the overall expression for transgenic arterial markers was normal in *rprml*-MO injected embryos with *Tg*(*dll4:GFP*) and/or *Tg*(*flt0*.*8:RFP*) background (Fig. S6E–I). Given that Notch activation is crucial for HSC specification^22^, we examined the spatiotemporal pattern of Notch signaling in the VDA using the *Tg*(*Tp1:GFP*) transgenic zebrafish line^23^. This reporter line is characterized by a fluorescent red signal in cells which respond to Notch signal activation. In these assays, we detected fewer Notch^+^ cells in the VDA in both *rprml*-morphant and mutant embryos (Fig. S6J–L). This indicates that VDA-cells are not actively responding to Notch signaling, thus affecting HSC specification from the VDA. Collectively, these results suggest that *rprml* is required for definitive HSPCs specification at the VDA in the hemogenic endothelium.

### *rprml* is required for the formation of the CHT

**Figure 4:**
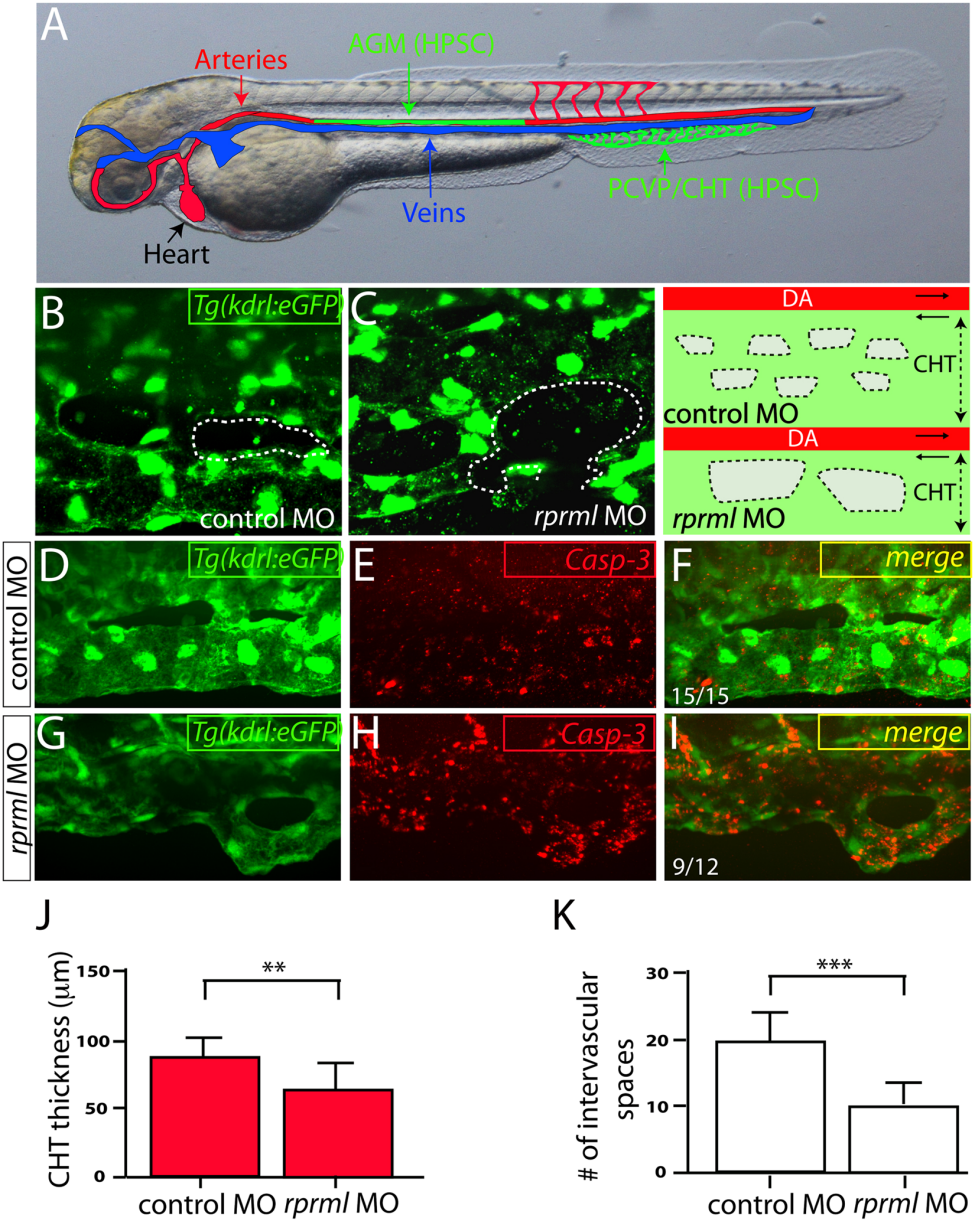
Lack of *rprml* increases cell apoptosis within the HSPC niche at the CHT. (**A**) Schematic diagram showing zebrafish vasculature at 48 hpf. In green: (1) aorta-gonad-mesonephros (AGM) where the HSPCs are born, and (2) the posterior caudal vein plexus/caudal hematopoietic tissue (PCVP/CHT), which constitutes the embryonic HSPC niche. Red and blue colors correspond to the artery and vein, respectively. (**B**–**I**) Confocal spinning-disk images of the CHT of *Tg*(*kdrl:eGFP*) at 48 hpf. (**B**,**C**) Note that the spaces are slightly larger in *rprml* morphants compared to control embryos. Right panel shows a schematic representation of the observed phenotypes from (**B**,**C**). Dotted areas indicate intervascular spaces within the CHT. (**E**,**F**,**H**,**I**) Anti-activated caspase-3 (Casp-3, red) labels apoptotic cells. (**D**,**F**) A control MO-injected embryo showing (**D**) normal morphology of the CHT and (**E**,**F**) standard levels of Casp-3 activity. (**G**–**I**) *rprml*-MO injected embryos show (**H**,**I**) increased Casp-3 immunoreactivity within the PCVP/CHT. Number of embryos with the phonotype shown as a fraction of the total number of embryos examined is indicated in the bottom left corner in (**F**,**I**). (**J**,**K**) Statistical significance was determined using two-tailed unpaired Student’s *t*-test. **P ≤ 0.01, ***P ≤ 0.001.

To explore if *rprml* has a function during HSPC niche formation, we studied CHT morphogenesis in *rprml*^*kd*^ double transgenic *Tg*(*flk:mcherry;fli1:eGFP*) zebrafish embryos. *rprml*^*kd*^ showed reduced thickness of the HSPC niche territory compared with wild type siblings (Fig. 4 and S7). Specifically, *rprml*^*kd*^ embryos displayed increased intervascular spaces at the posterior caudal vein plexus (PCVP; the network of vessels adjacent to the CHT; Fig. 4B,C,J–K and S7). To determine whether the cellular defects of the CHT in *rprml*^*kd*^ were due to increased apoptosis, we monitored cell death by using activated caspase-3 (Casp-3) immunostaining. Surprisingly, PCVP/CHT apoptosis in *rprml*^*kd*^ animals was increased (Fig. 4E,F,H,I). The significant difference in apoptosis between knockdown embryos and wild type/control siblings by 48 hpf, indicates that *rprml* is required for cell survival during the formation of the CHT. This finding partly explains the reduction in the HSPC niche by *rprml* loss-of-function. Alternatively, lack of *rprml* function may inhibit proper sprouting angiogenesis in the PCVP, thus hindering the establishment of proper sprouting connectivity by ECs at the PCVP/CHT. These mechanisms need to be further explored in the future.

## Discussion

Our previous studies found that *RPRM* family genes are expressed in the vasculature in zebrafish and humans, specifically in vascular smooth muscle cells and ECs^10^. Although a potential role of *RPRML* in human hematopoiesis has not been yet assessed, the recent association between the loss of *RPRM* and highly aggressive acute myeloid leukemia (AML)^15^ raises the question of whether products from the *RPRM* gene family play a relevant role during hematopoiesis. Here we provide the first report of a functional role of *rprml* in physiology, specifically during hematovascular development. Our results suggest that *rprml* is dispensable for primitive hematopoiesis but essential for the definitive wave of hematopoiesis. In absence of *rprml* activity, key hemangioblast genes such as *lmo2*, *fli1a* and *tal1/scl1* are normally expressed, indicating that *rprml* does not participate at early stages of hemangioblast specification in the ALM and PLM. In contrast, *cmyb*^+^ HSPC expression is strongly reduced in the VDA at the PBI (Fig. 1G–J) suggesting that *rprml* is essential for the activation of HSPC production. Together, these findings strongly suggest that *rprml* is not required for the initiation of hematopoiesis such as the primitive wave in the ICM but essential for definitive hematopoiesis along the VDA and PBI.

Previous studies have reported that MOs might present off-target RNA interactions at high concentrations. Accordingly, we followed the Stainier *et al*. guidelines^18^ for the use of MO in zebrafish: (i) we used both MOs and CRISPR-Cas9 gene-knockdown methods, (ii) we performed p53 qRT-PCR in *rprml* MO-injected embryos to discard potential toxicity^19,20^, and (iii) we performed rescue experiments to assess the specificity of the MOs. Notably, recent evidence has shown that MOs can reveal gene functions concealed by mutants^20^. Indeed, stable mutations induced by CRISPR-Cas9 might lead to gene compensation by up-regulation of other genes in the same signaling pathway. In contrast, MOs do not trigger as much compensation, therefore producing more extreme phenotypes than their mutant counterparts^20^. This allows for probing of gene function that is not possible using mutants alone. Our results indicate that our *rprml* MOs do not produce considerable off-target effects and the observed phenotype between morphants and CRISPR-Cas9 G0 genetic approaches are comparable. Therefore, gene knockdown by MOs is suitable to assess *rprml* gene function.

We also found that *rprml* deficiency drastically compromised HSPC specification from the VDA and the formation of the PCVP at 30–36 hpf, when budding of the HSPC from the artery floor occurs. The PCVP or HSPC niche appear incompletely branched at a critical period to receive the first HSPCs derived from the AGM/VDA. Initial formation of HSPCs from the VDA is significantly reduced, as characterized by a drastic reduction of *CD41:GFP*^*low*+^ HSPCs. Hence, we favor the hypothesis that reduction in HSPCs is due to a defect in the specification and formation of the HSPC perivascular niche at the CHT. We tracked the defect in CHT niche formation to increased apoptosis in *rprml*^*kd*^ embryos, as concluded by increased Casp-3 activity. This is consistent with results previously published in a conference paper^24^, which reported increased apoptosis secondary to knockdown of *RPRML* in human cultured cells. Theoretically, *rprml* knockdown could cause instability of HSPCs through increased apoptosis. Interestingly, overexpression of *RPRM* causes increased apoptotic activity^25,26^. Therefore, *RPRML* might have an opposing role when compared with *RPRM*^24^. All these possibilities need to be thoroughly examined in future research efforts.

During transient-definitive hematopoiesis, EMPs give rise to the first granulocytes produced in the PBI of the zebrafish embryos^5^. Our data show that generation of EMPs is impaired by *rprml* loss-of-function. *tal1/scl1*^+^*-*erythroid-, *pu*.*1*^+^-myeloid precursor- and early *mpx*^+^-granulocyte (including neutrophils)- cells fail to form in the PBI of *rprml*-deficient zebrafish embryos. Thus, *rprml* seems required for both waves of definitive hematopoiesis: formation of EMPs and HSPCs in the PBI and VDA, respectively. Whether this finding represents a conserved feature of vertebrates will have to be evaluated by future studies using cell culture and murine models. However, the retention of *rprml* in all main groups of vertebrates (e.g. tetrapods, coelacanths, bony fish, cartilaginous fish)^11^, could be an indication of the importance of its role in hematopoiesis in all vertebrate groups. In agreement with the phyletic distribution of *rprml*, other genes that are fundamental for hematopoiesis (e.g. *cmyb*) are also present in all main groups of vertebrates^27^.

In conclusion, the data presented here demonstrate for the first time a physiological role for the *rprml* gene. Specifically, we showed that *rprml* plays an essential role during both waves of definitive hematopoiesis by regulating the formation of EMPs and HSPCs. Additionally, *rprml* is required for proper HSPC niche formation in the PCVP/CHT. To our knowledge, this is the first study that provides an understanding of *rprml* function during hematopoiesis. Thus, the identification and validation of *rprml* as a regulator of definitive hematopoiesis may lead to a better understanding of both human physiology and pathology. Future studies of the physiological roles and functional interplay between the other members of the *Reprimo* gene family will shed light not only about the biological process in which the different *RPRM* genes are involved, but also of the functional evolutionary specification of genes that were originated as a product of whole genome duplications.

## Materials and Methods

### Zebrafish lines

Wild type TAB5 and transgenic strains were bred according to standard methods^28^. Embryos were raised in system water at 28 °C and staged according to either hpf or morphological criteria^29^. Transgenic lines used in this study correspond to *Tg*(*fli1:GFP;flk1:mCherry*), *Tg*(*flt0*.*8:mcherry;CD41GFP*), *Tg*(*flt0*.*8:RFP*), *Tg*(*fli1:GFP*)^28^, *Tg*(*kdrl:eGFP*), *Tg*(*Cd41:GFP*)^9^, *Tg*(*dll4:GFP*), *Tg*(*Tp1:nRFP*)^23^ and *Tg*(*mpx:GFP*)^29^. All zebrafish studies and experimental protocols were conducted under the guidance and approval of the Institutional Animal Care and Use Committee and Bioethical Advisory Board at Pontificia Universidad Católica de Chile; and followed the AALAC reference resource on guidance on the housing and care of zebrafish (*Danio rerio*)^30^.

### Knockdown of *rprml* by use of CRISPR-Cas9 genetic system

We used the CRISPR-Cas9 system to disrupt *rprml*. CRISPR short guide RNAs (sgRNAs) targeting the *rprml* exon 1 (the only exon present in *rprm* gene family) were designed using open access internet-based software CRISPRscan (http://www.crisprscan.org/)^31^. This software was used to determine an on-target score to predict Cas9 cleavage efficiency using previously described algorithms^31^. sgRNAs were tested *in vitro* for efficiency (New England Biolabs: 10.17504/protocols.io.ch2t8d). Using the sgRNA forward primer for T7 template 5′-TAATACGACTCACTATAGGTAGGACTGAGTTAGCCGCGTTTTAGAGCTAGAA-3′ and the invariance reverse primer 5′-AAAAGCACCGACTCGGTGCCACTTTTTCAAGTTGATAACGGACTAGCCTTATTTTAACTTGCTATTTCTAGCTCTAAAAC-3′. Next, fertilized zebrafish eggs from the transgenic line *Tg*(*fli1:GFP*) were injected with a mixture of Cas9 protein (400 ng/µL) and gene-specific sgRNAs (300 ng) at one-cell developmental stage, as previously described^31^. To identify indel mutations in injected embryos with vascular defects, we used the T7 endonuclease assay (Fig. S2F)^32,33^. This assay relies on the detection of single-base mismatches in the target DNA sequence. A 20 μL reaction volume containing 300 ng of each target DNA amplified from genomic DNA of microinjected and wild type embryos and 1 μL of T7 endonuclease (New England Biolabs) were incubated at 37 °C for an hour. Results were observed on a 1.5% agarose gel. The *rprml* primer sequences used for T7 endonuclease were; forward primer (5′-3′): CTCCCTCCATCCATCCATGC, and reverse primer (5′-3′): GAACCTCTCGTCCACATCGG.

### Morpholino and RNA injections

Embryos were microinjected at 1–2 cell-stage with 3 nL of morpholino (MO) solution (along with phenol red) at a concentration of 0.5 mM for *rprml* and 1 mM for the control MOs. MOs were obtained from Gene Tools (Gene Tools, LLC) and designed to block the translation start site. MO sequences were: *rprml* MO (5′-3′) = ACGTTCCGTTCATCCTGAGCAGAGA, and control MO (5′-3′) = CCTCTTACCTCAGTTACAATTTATA. Since *rprml* is a single-exon gene, it is not possible to design MOs targeting the splicing donor sites. Efficiency of knockdown was assessed by reduction in protein synthesis, as confirmed by immunohistochemistry (IHC) staining in *rprml* morphants compared to control MO-injected embryos (Fig. S2D,E). This antibody was validated in our previous work^14^. Messenger RNA (mRNA) was co-injected with MOs in phenotypic rescue experiments (Fig. S5).

### RPRMs molecular cloning and rescue experiments

*Rprml* gene was amplified by PCR by using the following primer sequences Fwd (5′-3′) = AGTTGCTCGACTCAACAGGC and Rv (5′-3′) = TGTTAACAGGTGTGACCCGC. PCR products were gel purified by using AxyPrep™ DNA Gel Extraction Kit (Axygen) and cloned into pCRII-TOPO cloning vector (Invitrogen) according to manufacturer´s instructions. cDNA fragment identity was confirmed by sequencing. In order to generate mRNA, *rprml* was subcloned into pCS2+ plasmid vector by using BamHI and XhoI restriction enzyme sites. In turn, the plasmid was linearized by using NotI and messenger RNA was obtained by using mMessage-mMachine kit (Ambion) according to manufacturer’s instructions.

### RNA extraction and cDNA synthesis from zebrafish embryos

For cDNA synthesis, total RNA was collected from pools of 50 *rprml* MO-injected and 50 uninjected control embryos at 48 hpf using E.Z.N.A total RNA kit I (OMEGA), according to manufacturer’s instructions. cDNA was synthesized from 1 µg of total RNA with oligo (dT) using ImProm-II Reverse transcriptase II (PROMEGA) following manufacturer’s guidelines.

### Quantitative polymerase chain reaction (qPCR)

Quantitative (q) PCR was carried out with a Stratagene Mx3000P detector system (Agilent Technologies) with optic tubes (SSI Innovations for Life Science) using 2× Brilliant II SYBR® Green qPCR Master Mix (Agilent Technologies). Four technical replicates were performed for *rprml* MO-injected embryos and three were performed for uninjected control embryos. *actb1* was used as reference gene in all qRT-PCR reactions. *actb1* forward primer (5′-3′) = TGAGCAGGAGATGGGAACC, and reverse primer (5′-3′) = CAACGGAAACGCTCATTGC (product size 102 base pairs -bps-). *p53* forward primer (5′-3′) = CAGTCTGGCACAGCAAAATC, and reverse primer (5′-3′) = TTTGCCAGCTGACAGAAGAG (product size 74 bps). Gene expression data was processed by MxPro qPCR Software 4.10. Relative p53 expression levels in *rprml* MO-injected embryos and controls are expressed as ΔCq values:$\,{\rm{\Delta }}\mathrm{Cq}\,={\rm{Cq}}({\rm{reference}})\mbox{--}{\rm{Cq}}({\rm{target}})$. Graphical representation of the data are shown as mean ΔCq ± (SEM). All relevant qPCR data are available in the supplementary material (Supplementary Table 1).

### Whole mount *in situ* hybridization

Templates for probe synthesis were amplified from embryonic zebrafish cDNA using primers including T7 RNA polymerase promoter sequence. The 5′ untranslated regions (5′-UTR) of *rprml* gene was used for primer design. Expression pattern analysis of hematopoietic (*tal1/scl1*, *pu*.*1*, *mpx*, *cmyb*), vascular (*fli1*, *lmo2*) and T-lymphocyte (*rag-2*) markers was carried out as described previously^21,34,35^. Purified PCR products were transcribed *in vitro* and labeled using digoxigenin (DIG) RNA labeling Kit (Roche) according to manufacturer’s instructions. Riboprobes were purified by mini Quick Spin RNA Columns (Roche) and stored at −80 °C with deionized formamide for posterior use. Whole mount *in situ* hybridization (WISH) was carried out as previously described^36^.

### Immunohistochemistry (IHC) staining

24 to 48 hpf embryos were fixed overnight in 4% paraformaldehyde at 4 °C and dehydrated in methanol for subsequent use. Rehydrated embryos were treated with acetone for 20 minutes at −20 °C, washed and treated with Proteinase K 10 ng/mL for 15 min as antigen exposure steps. Blocking was carried out with 10% FBS + 1% DMSO in PBT twice for 1 hour each. Embryos were incubated with the primary antibody overnight at 4 °C in blocking solution at corresponding dilution. Rabbit polyclonal anti-activated Caspase-3 antibody (Abcam, ab13847) was used at a 1:500 dilution, mouse monoclonal anti-GFP at 1:1000 dilution (Thermo Fisher Scientific, A11120), rabbit polyclonal anti-GFP at 1:500 dilution (Thermo Fisher Scientific, A11122) and rabbit polyclonal anti-RPRM at 1:1000 dilution (USbiological life science, 364937). This polyclonal antibody recognizes a common sequence for RPRM and RPRML, justifying its use for assessment of Rprm/Rprml protein expression in *rprml*-morphant embryos. For secondary antibody labeling, goat anti-rabbit Alexa-555, goat anti-mouse Alexa 488, goat anti-mouse Alexa 488, and goat anti-rabbit Alexa 546 (Invitrogen) were used at a 1:200 dilution in blocking buffer for 2 hours at room temperature.

### Image acquisition, processing and quantification

WISH stained zebrafish embryos were embedded in 75% glycerol/PBS and imaged using a NIKON eclipse 80i microscope equipped with a DS-Vi1 (NIKON) camera. For whole-mount fluorescent IHC images, embryos were mounted in acrylic rings with glass bottoms containing 1% low melting point agarose and images were acquired using a Zeiss LSM780 laser-scanning spinning disc microscope. VE-DIC and fluorescent microscopy were performed as described previously^37^ through the 60X/1.00 water-immersion objective of a Nikon eclipse E200 microscope with a 519CU 5.0 M CMOS Camera using the Micrometrics SE Premium 4 program. To quantify the effect of the morpholinos on the development of the vascular system, CHT thickness was measured using ImageJ64. Pooled data were calculated as the mean plus or minus SD with minimum number of three independent experiments. Pairwise comparisons were subject to statistical analysis using unpaired two-tailed Student’s *t*-test. P-value < 0.05 was considered statistically significant.

## Supplementary information

Supplementary Information.pdf

Supplementary Table 1
