# Author Correction: The Reprimo gene family member, *reprimo-like* (*rprml*), is required for blood development in embryonic zebrafish

**DOI:** 10.1038/s41598-020-59857-9

**Published:** 2020-02-28

**Authors:** Karen Stanic, German Reig, Ricardo J. Figueroa, Pedro A. Retamal, Ignacio A. Wichmann, Juan C. Opazo, Gareth I. Owen, Alejandro H. Corvalán, Miguel L. Concha, Julio D. Amigo

**Affiliations:** 1 Departamento de Fisiología, Facultad de Ciencias Biológicas, Pontificia Universidad Católica de Chile, Santiago, Chile; 2 Institute of Biomedical Sciences, Faculty of Medicine, Universidad de Chile, Santiago, Chile; 3 Advanced Center for Chronic Diseases (ACCDiS), Santiago, Chile; 4 Laboratorio de Oncología, Departamento de Hematología y Oncología, Facultad de Medicina, Pontificia Universidad Católica de Chile, Santiago, Chile; 5 Instituto de Ciencias Ambientales y Evolutivas, Facultad de Ciencias, Universidad Austral de Chile, Valdivia, Chile; 6 Millennium Institute on Immunology and Immunotherapy, Santiago, Chile; 7 Biomedical Neuroscience Institute, Santiago, Chile, Center for Geroscience, Brain Health and Metabolism, Santiago, Chile; 8 Universidad Bernardo O ´Higgins, Escuela de Tecnología Médica and Centro Integrativo de Biología y Química Aplicada (CIBQA), Santiago, Chile

Correction to: *Scientific Reports* 10.1038/s41598-019-43436-8, published online 09 May 2019

Ricardo J. Figueroa and Pedro A. Retamal were omitted from the author list in the original version of this article. This has been corrected in the PDF and HTML versions of the Article, and in the accompanying Supplementary Information file.

The Author Contributions section now reads:

“Conceptualization: G.R., I.A.W. and J.D.A. Investigation: K.S. and J.D.A. Methodology: K.S., I.A.W., R.J.F., P.A.R., and J.D.A. Editing and writing the manuscript: K.S., G.R., I.A.W., J.C.O., G.I.O., A.H.C., M.L.C. and J.D.A. Original draft, analysis and funding acquisition: J.D.A.”

The Acknowledgements section now reads:

“The authors wish to thanks to A. Siekmann, N. Lawson and B. Weinstein for providing the Tg(dll4:GFP), Tg(flt0.8:RFP), Tg(Tp1:GFP) and Tg(fli1:GFP;flk1:mcherry) transgenic line. We also wish to thank Dr. Jon D. Moulton for his insights and helpful comments on the manuscript. This work was funded by grants BMBF-CONICYT 20140027 to J.D.A., CONICYT-PIA ACT1402 to J.D.A. and M.L.C., CRP-ICGEB CH15-01 to J.D.A., FONDECYT 1160627 to J.C.O., FONDECYT 1180241 to G.I.O., FONDECYT 1151411 to A.H.C., CONICYT-FONDAP 15130011 to A.H.C. and G.I.O., CONICYT-FONDAP 15150012 to M.L.C., Millennium Science Initiative ICM P09-015-F to M.L.C. and IMII P09/016-F to G.I.O., FONDECYT 11170761 and PAI 79170126 to G.R.”

