# Supplementary material for: The Reprimo gene family member, reprimo-like (rprml), is required for blood development in embryonic zebrafish
Source: Sci Rep. 2019 May 9;9:7131. doi: 10.1038/s41598-019-43436-8 (PMC6509255; doi:10.1038/s41598-019-43436-8)
Supplement: Supplementary file 1 — Supplementary Information.pdf [file 41598_2019_43436_MOESM1_ESM.pdf]

Supplementary Information

Title: The Reprimo gene family member, *reprimo-like* (*rprml*), is required for blood development in embryonic zebrafish

Karen Stanic[1], German Reig[2], Ignacio A. Wichmann[3,4], Juan C. Opazo[5], Gareth I. Owen[1,3,6], Alejandro H. Corvalán[3,4], Miguel L. Concha[2,7], Julio D. Amigo[1*]

[1]Departamento de Fisiología, Facultad de Ciencias Biológicas, Pontificia Universidad Católica de Chile, Santiago, Chile. [2]Institute of Biomedical Sciences, Faculty of Medicine, Universidad de Chile, Santiago, Chile. [3]Advanced Center for Chronic Diseases (ACCDiS), Santiago, Chile. [4]Laboratorio de Oncología, Departamento de Hematología y Oncología, Facultad de Medicina, Pontificia Universidad Católica de Chile, Santiago, Chile. [5]Instituto de Ciencias Ambientales y Evolutivas, Facultad de Ciencias, Universidad Austral de Chile, Valdivia, Chile. [6]Millennium Institute on Immunology and Immunotherapy, Santiago, Chile. [7]Biomedical Neuroscience Institute, Santiago, Chile. Center for Geroscience, Brain Health and Metabolism, Santiago, Chile.

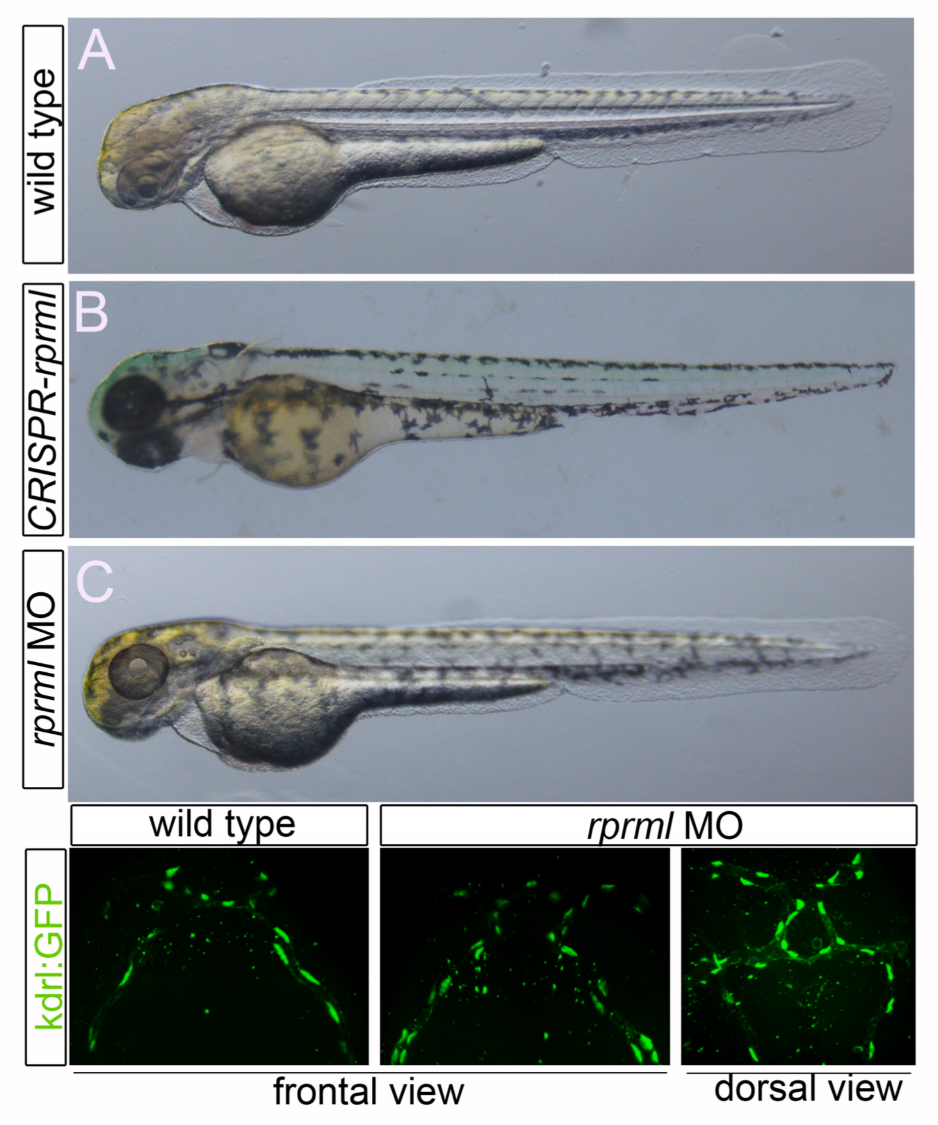

Figure S1: Bright-field microscopy shows no macroscopic anomalies in *rprml*-morphants/mutants. (A-C) Lateral view of live embryos by bright field microscopy at 48hpf. (A) wild type, (B) *CRISPR-Cas9-rprml* and (C) *rprml* MO-injected embryos reveals grossly normal morphology. (Bottom panel) Normal vascular development in the brain vessels of wild type and *rprml* morphant embryos.

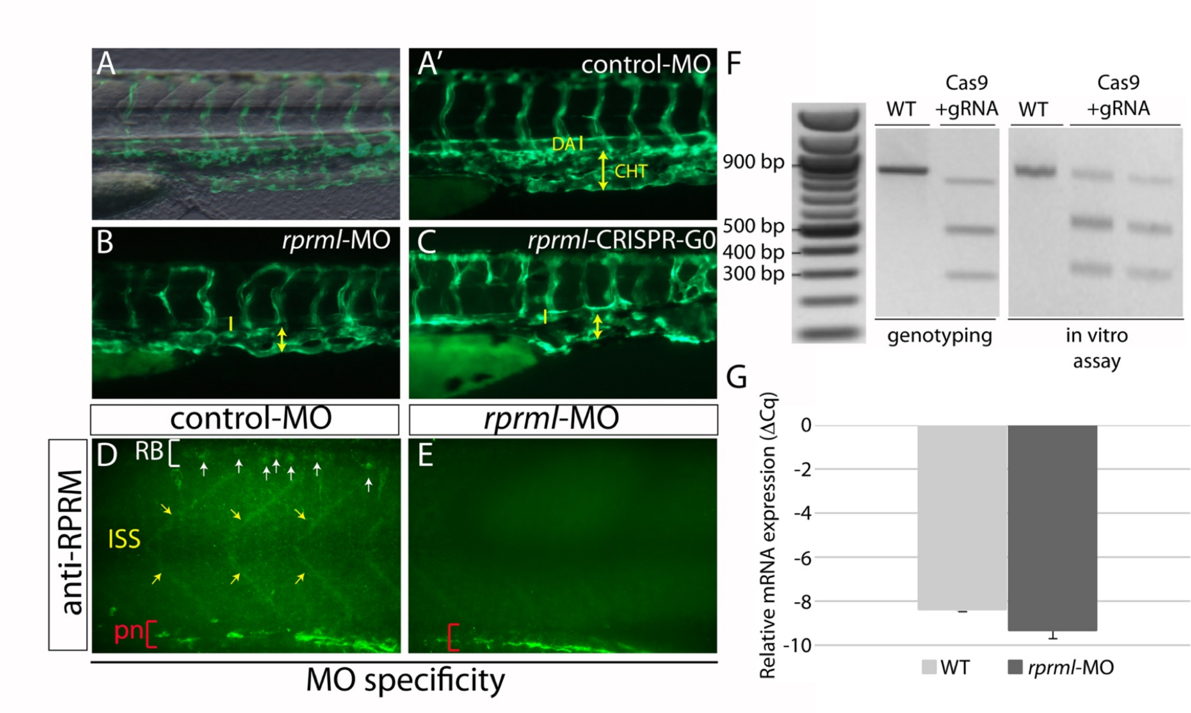

Figure S2: Similar phenotypes are obtained by disruption of *rprml*, either by CRISPR-Cas9 mutation or injection of antisense MOs. (A-E) lateral views of (A-C) live transgenic *Tg(fli1:GFP)* or (D-E) anti-RPRM IHC stained embryos at 48 hpf visualized by fluorescence and confocal microscopy, respectively. (A-A') Normal vascular morphology is observed in control-MO-injected embryos. (B-C) Embryos injected with MOs or CRISPR-Cas9 targeting *rprml* exhibit reduced caudal hematopoietic tissue (CHT) territory (yellow double arrows). The yellow line represents the dorsal aorta (DA). (D-E) Rprm/Rprml protein expression is effectively blocked by *rprml*-MO. A human anti-RPRM/RPRML antibody labeled the Rohon-Beard neurons (RB, white arrows), the inter-somitic spaces (ISS, yellow arrows) and the pronephric tubule (pn, red brackets). A drastic reduction in immunoreactivity is observed in *rprml* MO-injected embryos compared to control MO-injected embryos. (F) Left panel: *rprml* CRISPR-Cas9 embryo genotyped by T7 endonuclease assay shows INDEL mutations of the expected sizes (500bp, 350bp). Right panel: *in vitro* DNA cleavage activity assay showing cropped areas containing Cas9-cleaved DNA bands of the expected sizes for *rprml*. A 1 kb DNA ladder was used as a molecular weight marker. (G) qPCR showing p53 relative expression in wild type and *rprml* MO specimens. Data shown as ΔCq ± SEM, (CI 95% = -8.41 ± 0.135 for wild type controls; CI 95% = -9.36 ± 0,65 for *rprml* morphants).

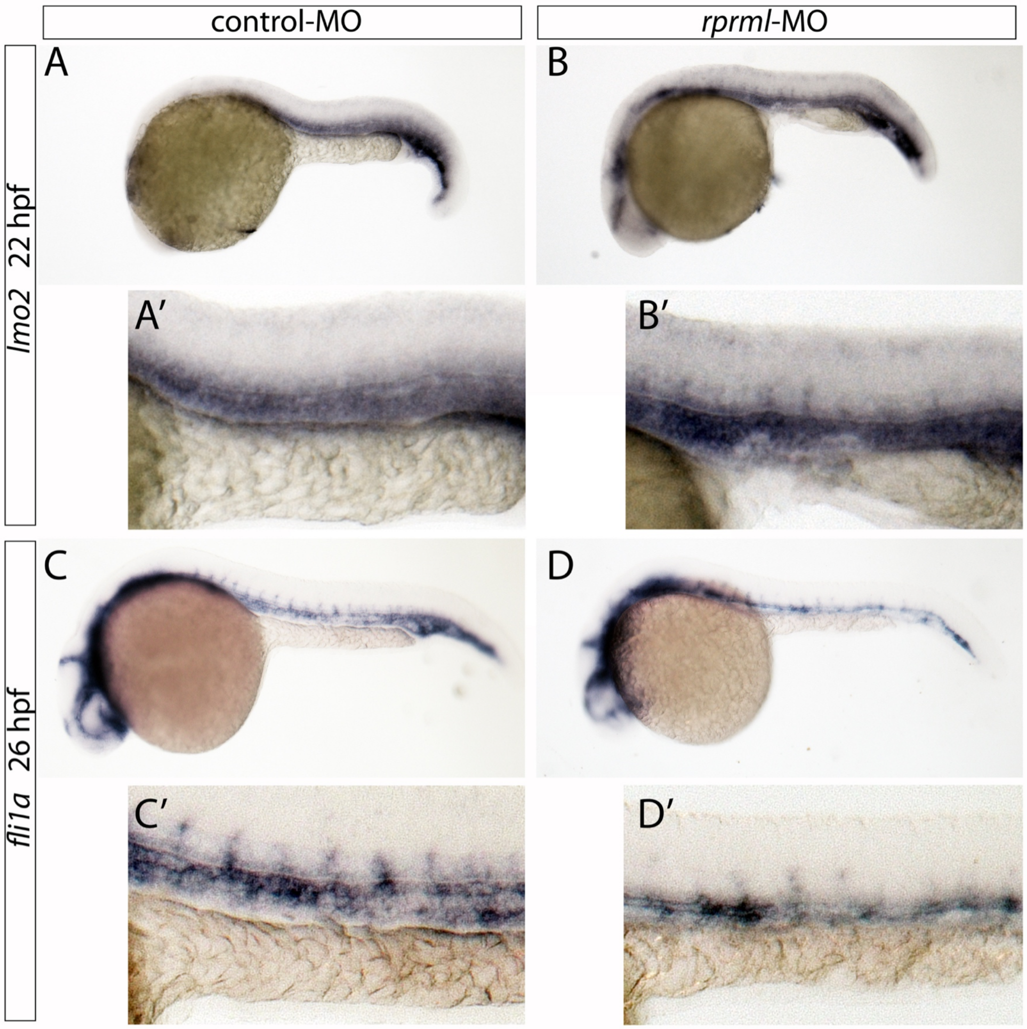

Figure S3: Early vascular development in *rprml* morphants. (A-D) Lateral view of embryonic zebrafish at 22 and 26 hpf analyzed by WISH for *lmo2* (A-B) and *fli1a* (C-D). (A'-D') Magnification of the trunk vessels.

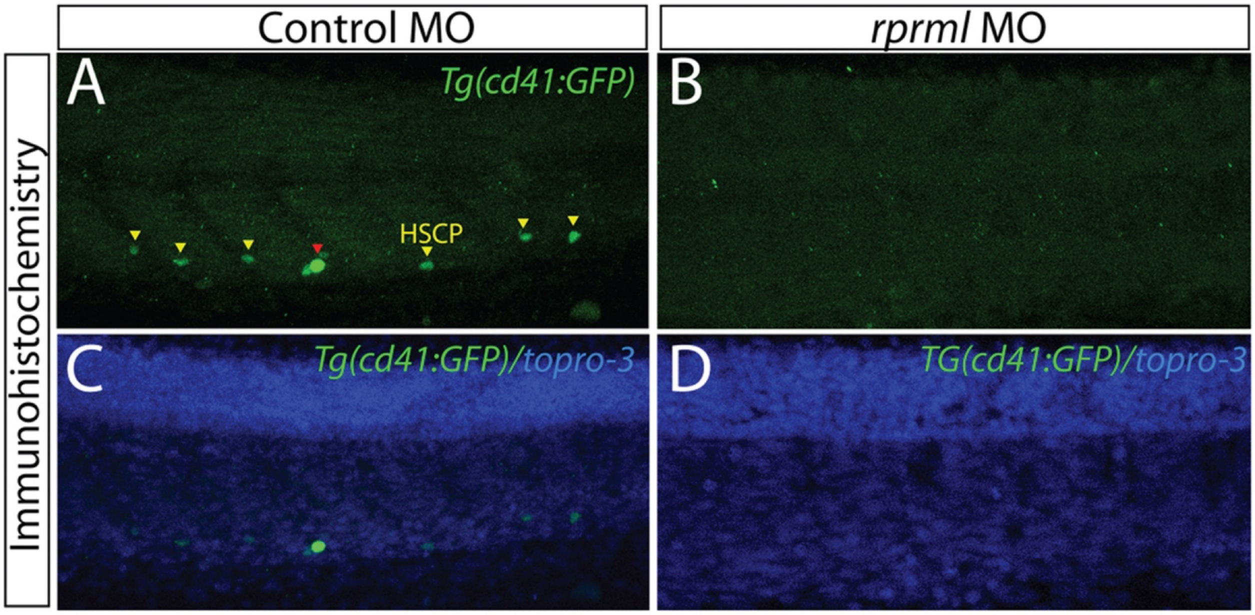

Figure S4: CD41 expression is reduced in the CHT of *rprml* morphants. (A-D) Lateral view of the trunk with anterior to the left. (A-B) Whole mount immunohistochemistry/immunofluorescence showing *Tg(CD41:GFP)* expression pattern at 54 hpf. Staining with anti-GFP indicates expression of HSPC (yellow arrows) in the CHT (green bracket). (C-D) *topro-3* staining (blue) shows the localization of the nuclei.

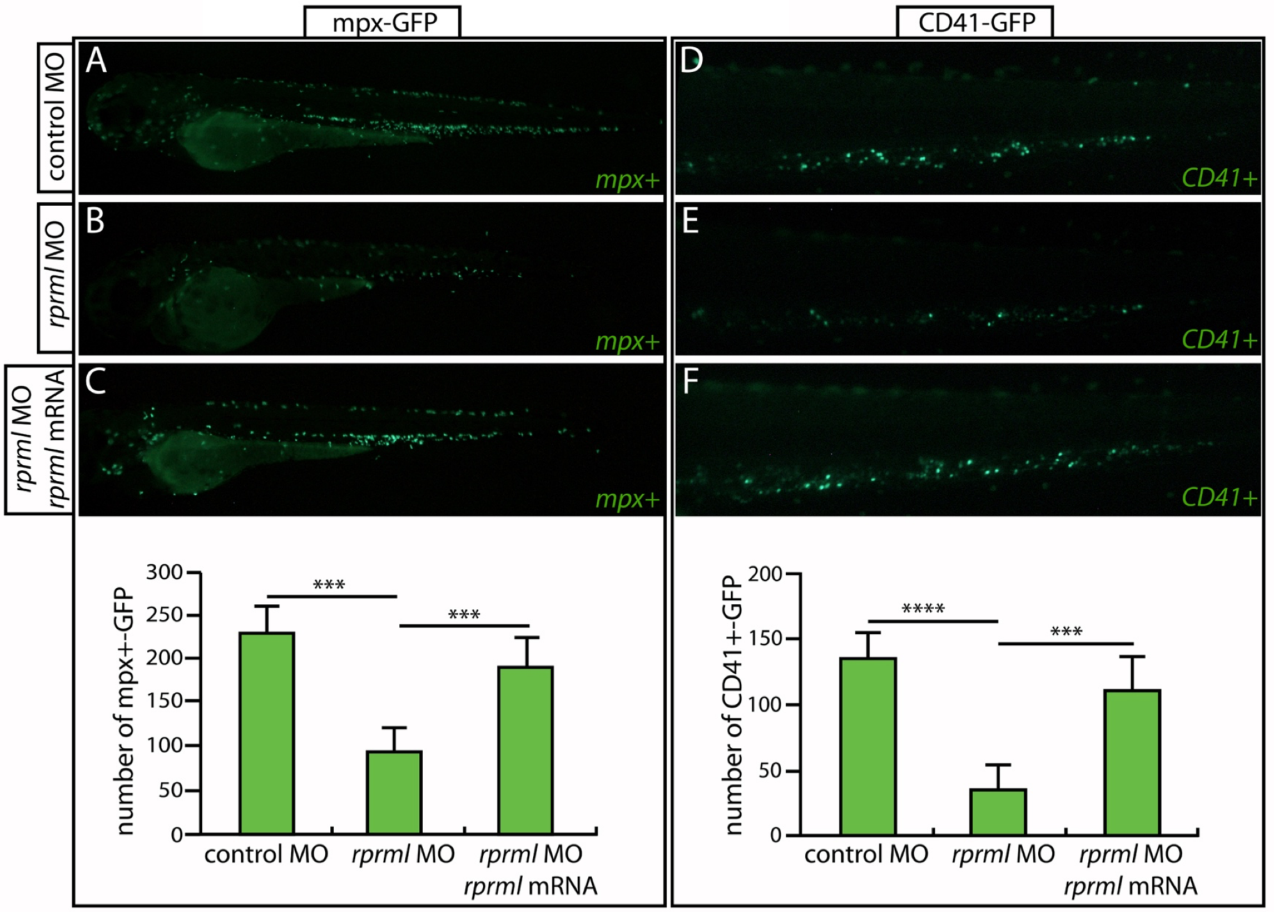

Figure S5: *rprml* deficiency by MOs results in reduce *mpx*+- and *CD41*+- cells during hematopoiesis. Fluorescent microscopy images for: (A-C) *mpx*+- and (D-F) *CD41*+ cell populations in transgenics *Tg(mpx:GFP)* and *Tg(CD41:GFP)* respectively . (B, E) *rprml*-MO injections cause reduction in *mpx*+- and *CD41*+ blood cells. (C, F) Co-injection *rprml*-MO and *rprml* mRNA significantly rescued the *rprml* morphant phenotype. (Bottom panels) Statistical significance was determined using two-tailed unpaired Student's *t*-test. *** $P \leq 0.001$, **** $P \leq 0.0001$.

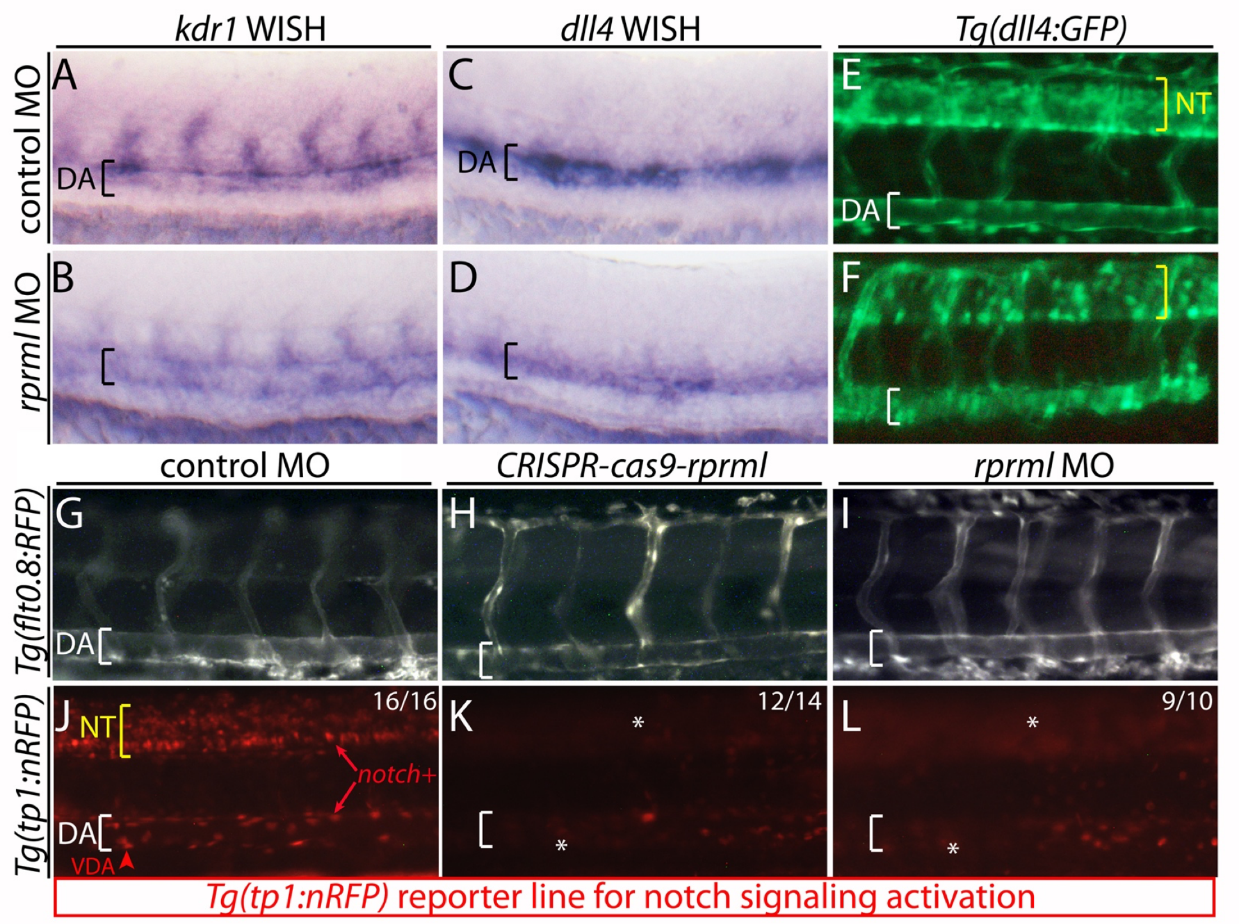

Figure S6: lack of *rprml* hinders normal activation of the Notch signaling pathway. (A-D) Lateral views of 24 hpf analyzed by WISH for (A-B) arterial endothelial cells (*kdr1*) and (C-D) vascular arterial progenitor cells (*dll4*). (E-L) Fluorescent microscopy images of transgenics: (E-F) *Tg(dll4:GFP)*, (G-I) *Tg(flt0.8:RFP)* and (J-L) *Tg(Tp1:nRFP*). Brackets indicated the positioning of the dorsal aorta (DA). (K-L) Asterisks indicated reduced Notch activity in *CRISPR-Cas9-rprml* and/or *rprml*-MO injected embryos (DA). The number of embryos with the phenotype shown as a fraction of the total number of embryos examined is indicated in the top right corner in J-L.

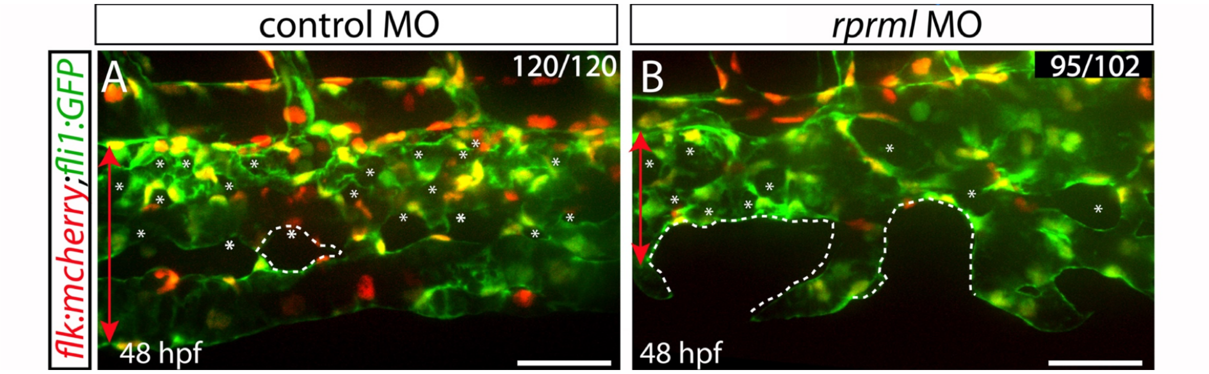

Figure S7: Knockdown of rprml impairs normal CHT niche morphogenesis. (A-B) Lateral view of double transgenic *Tg(flk:mcherry;fli1:eGFP)* embryos microinjected at 1-2 cell-stages with (A) control and (B) *rprml* morpholinos (MOs). The visualization at 48 hpf was done by live longitudinal confocal microscopy. Double arrows show the girth of the caudal hematopoietic tissue (CHT). Asterisks represent intervascular spaces. The number of embryos with the phenotype is shown as a fraction of the total number of examined embryos, indicated in the top right corner in A-B. Scale bars 50 μm.
